# A dynamic flow model mimicking duodenoscope reprocessing after bacterial contamination for translational research

**DOI:** 10.1017/ash.2022.294

**Published:** 2022-09-13

**Authors:** Maarten Heuvelmans, Willem Woudstra, Herman F. Wunderink, Jan F. Monkelbaan, Henny C. van der Mei

**Affiliations:** 1 Department of Medical Microbiology, University Medical Center Utrecht, Utrecht, The Netherlands; 2 Department of Biomedical Engineering, University of Groningen, Groningen, The Netherlands; 3 Department of Gastroenterology and Hepatology, University Medical Center Utrecht, Utrecht, The Netherlands

## Abstract

**Objective:**

Duodenoscopy-associated infections and outbreaks are reported globally despite strict adherence to duodenoscope reprocessing protocols. Therefore, new developments in the reprocessing procedure are needed.

**Design:**

We evaluated a novel dynamic flow model for an additional cleaning step between precleaning and manual cleaning in the reprocessing procedure.

**Methods:**

A parallel plate flow chamber with a fluorinated ethylene propylene bottom plate was used to mimic the duodenoscope channels. The flow chamber was inoculated with a suspension containing *Klebsiella pneumoniae* to simulate bacterial contamination during a duodenoscopic procedure. After inoculation the flow chamber was flushed with a detergent mimicking precleaning. Subsequently the flow chamber was subjected to different interventions: flow with phosphate-buffered saline (PBS), flow with 2 commercial detergents, flow with sodium dodecyl sulfate with 3 different concentrations, and flow with microbubbles. Adhering bacteria were counted using phase-contrast microscopy throughout the experiment, and finally, bacterial viability was assessed.

**Results:**

During precleaning both PBS and 1% (v/v) Neodisher Mediclean Forte were able to desorb bacteria, but neither proved superior. After precleaning only sodium dodecyl sulfate could desorb bacteria.

**Conclusions:**

Flushing during precleaning is an essential step for reducing adhering luminal bacteria, and sodium dodecyl sulfate is a promising detergent for bacterial desorption from duodenoscope channels after precleaning.

Duodenoscopy-associated bacterial infections and outbreaks occur globally, despite strict adherence to duodenoscope reprocessing protocols.^1^ Contamination of duodenoscopes after reprocessing occurs frequently, with rates as high as 15% resulting in 32 reported outbreaks between 2000 and 2017 worldwide with almost 400 affected patients.^1,2^ Clearly the risk of bacterial contamination of duodenoscopes is not eliminated entirely by current reprocessing protocols, and additional measures are needed to further optimize the reprocessing procedure. In 2015, to reduce the risk of duodenoscopy-associated pathogen transmission, the US Food and Drug Administration advised 4 supplemental reprocessing measures, including double high-level disinfection, a culturing and quarantine program, ethylene oxide gas sterilization, and liquid chemical sterilization.^3^ Unfortunately, these supplemental reprocessing measures have been shown to lack efficacy and are not cost-effective.^4–10^ Therefore, duodenoscopy-associated transmission remains a problem and development of novel effective reprocessing techniques is needed.

Given the high costs of duodenoscopes, it is not feasible to use them for testing, and development of a universal and practical test model will be required. Several models mimicking bacterial contamination and evaluating the reprocessing of duodenoscopes with various designs have been described.^11–14^ Some models used a static system instead of a flow system. They do not resemble repeated passage of fluids through the duodenoscope channel and do not allow for evaluation of reprocessing methods using flow.^11,12^ To resemble the lumen of a duodenoscope with flow, models using polytetrafluoroethylene (PTFE) tubing can be used.^13,14^ In this study, we developed a parallel plate flow chamber with flow control and real-time visualization of bacterial adhesion.^15,16^ This model is unique because it combines the hydrophobic properties of PTFE by using fluorinated ethylene propylene (FEP) while allowing for real-time visualization.

We evaluated a novel dynamic flow model for an additional transport cleaning step between precleaning and manual cleaning in the reprocessing procedure. The interventions were envisioned during transport so the entire reprocessing procedure is not lengthened. If this additional step proves to be effective, then further research could be focused on optimizing the complete process for instance by replacing precleaning and/or (part of) manual cleaning (Fig. 1). We evaluated 7 different interventions for their ability to desorb adhering luminal bacteria. The following interventions were tested in this study: flow with phosphate buffered saline (PBS); flow with 2 commercial reprocessing products; flow with sodium dodecyl sulfate (SDS) 1%, 2%, or 5%; and flow with microbubbles.

**Fig. 1.**
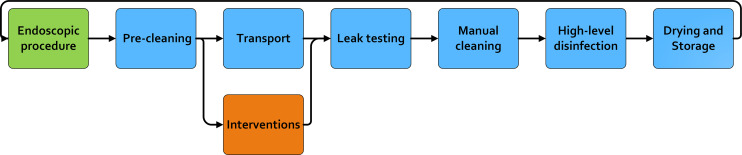
The current steps of duodenoscope reprocessing.^32^ Green block: the duodenoscopic procedure; blue blocks: current steps of the reprocessing procedure; orange block: additional step evaluated in this study.

## Methods

### Bacterial growth conditions and harvesting

A *Klebsiella pneumoniae* strain isolated from a duodenoscope during an outbreak was used to inoculate the flow chamber.^17^ The strain was cultured from a frozen stock on blood agar (tryptone soya agar with 5% sheep blood, Mediaproducts, Groningen, The Netherlands) and was aerobically incubated for 24 hours at 37°C. One colony was inoculated in 10 mL tryptone soya broth (TSB, Oxoid, Basingstoke, UK) and incubated for 24 hours at 37°C. Subsequently, 10 mL bacterial suspension was added to 200 mL TSB and incubated for 16 hours at 37°C. Bacteria were harvested by centrifugation at 5,000×*g* and washed twice with PBS (10 mM potassium phosphate and 150 mM sodium chloride; pH, 7.0). The bacterial pellet was resuspended in PBS supplemented with 2% (v/v) TSB to a final concentration of 10^9^ bacteria per milliter, as determined by enumeration with a Bürker-Türk counting chamber.

### The parallel plate flow chamber as a model for the duodenoscope

The parallel plate flow chamber (175 × 17 × 0.75 mm^3^) and image analysis system have been described previously.^15,16^ The top plate of the flow chamber was made from glass and the bottom plate was covered with a 25-μm FEP sheet (Holscot Europe, Grantham, UK) to mimic the hydrophobic lumen of a duodenoscope channel (Appendix 1). Transparent hydrophobic FEP was used instead of nontransparent hydrophobic PTFE used in duodenoscopes to evaluate bacterial adhesion in real time in the flow chamber while retaining the same hydrophobic properties as PTFE. Images of bacterial adhesion and desorption were taken with a charge-coupled device camera mounted on a phase-contrast microscope. Prior to the assembly of the flow chamber, all components were extensively cleaned with detergent, water, methanol, and water again as a final step. Flasks containing bacterial suspension and buffer were positioned at a higher elevation with respect to the chamber to ensure circulation of fluids by hydrostatic pressure. Constant flow was maintained by recirculation of the fluids using a roller pump.

### Bacterial adhesion and desorption during different interventions

Prior to each experiment, the flow chamber was filled with PBS to remove all air bubbles in the system. Subsequently, the flow chamber was filled with bacterial suspension (1 × 10^9^ bacteria/mL). The flow was switched off and the bacterial suspension was allowed to remain in the chamber for 30 minutes to simulate continued contamination during a gastrointestinal procedure. After 30 minutes, 75 mL PBS was passed through the flow chamber at a flow rate of 450 mL per minute to remove the bacterial suspension and nonadhering bacteria. Subsequently, images were taken from12 fixed points on the bottom plate of the flow chamber prior to and after the interventions. Adhering bacteria in these images were automatically counted using ImageJ version 1.49 software (Fiji National Institutes of Health, Bethesda, MD).^18^ The mean of the 12 images was calculated and the difference between the mean prior to and after an intervention was calculated as follows:
(1)

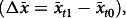




in which *x̄*_*t*0_ was the mean number of bacteria/cm^2^ prior to precleaning or intervention and *x̄*_*t*1_ was the mean number of bacteria/cm^2^ after precleaning or intervention.

To simulate precleaning of a duodenoscope, the flow chamber was flushed with 25 mL PBS or 1% (v/v) Neodisher Mediclean Forte (NDMCF, Dr Weigert, Hamburg, Germany) at a flow rate of 450 mL per minute. After flushing, the bottom plate of the flow chamber was imaged to determine the number of bacteria that desorbed from the bottom plate during the precleaning step. Only experiments where the flow chamber was flushed with NDMCF were subjected to further intervention, in accordance with the current reprocessing protocol of duodenoscopes in the University Medical Center Utrecht. After the precleaning step with PBS or NDMCF the flow chamber was exposed to different interventions. These interventions were all applied for 2 hours at a flow rate of 14 mL per minute and included flow with PBS; flow with 1% (v/v) NDMCF; flow with 20 g/L Neodisher Septo Active (NDSA, Dr. Weigert); flow with 1%, 2%, or 5% (w/v) SDS (Bio-Rad Laboratories, Hercules, CA); and flow with microbubbles. Microbubbles were generated by running 8 cycles of 9 minutes with the Braun OxyJet type 3721 (Proctor & Gamble, Cincinnati, OH) on the fifth setting. As a control, inoculated flow chambers that had been subjected to an NDMCF flush during precleaning were drained and left with residual moisture for 2 hours.

Following the interventions, images were taken from the bottom plate, after which the flow chamber was disassembled and the FEP recovered to determine bacterial viability. The FEP was cut in 3 equal sections, and bacterial viability was determined using the 3M Petrifilm aerobic count (3M, Saint Paul, MN). The FEP pieces were placed into the hydrated Petrifilm and incubated for 48 hours at 37°C. After 48 hours, the number of colony-forming units (CFU) were counted. In total, every intervention was performed at least 3 times.

### Determination of the minimal inhibitory and bactericidal concentration

The minimal inhibitory concentration (MIC) and minimal bactericidal concentration (MBC) of the different detergents used for the interventions were determined using serial dilutions in a 96-well round-bottom plate (Costar, Corning, Corning, NY). Detergents were 2-fold serially diluted in demineralized water after which 100 μL bacterial suspension (2 × 10^5^ bacteria/mL) was added in double-concentrated TSB. The final bacterial concentration was 1 × 10^5^ bacteria/mL. The 96-well plate was incubated aerobically for 24 hours at 37°C and was then evaluated for visual growth. The first well without visual growth was the MIC. From each well without visual growth, 10 µL bacterial suspension was cultured on a blood agar plate and incubated aerobically for 24 hours at 37°C. The lowest concentration with no growth on the blood agar plate was regarded as the MBC. The determinations of the MIC and MBC were performed in triplicate with separately cultured bacteria.

### Statistical analysis

All statistics were calculated using GraphPad Prism version 8.3.0 software (GraphPad Software, San Diego, CA) and *P <* .05 was considered significant. An unpaired *t* test was used to compare the PBS and NDMCF flush during simulated precleaning. The interventions were compared to the flow chambers without intervention and analyzed using separate unequal variance *t* tests. To analyze the results of the viability assay, growth was divided into 3 categories: <30 CFU/cm^2^, 30–300 CFU/cm^2^, and >300 CFU/cm^2^.^19^ These categorical data were analyzed by comparing the viability from flow chambers with and without intervention with separate Mann-Whitney *U* tests.

## Results

In this study, bacterial desorption in a duodenoscope reprocessing dynamic flow model was investigated. Both precleaning and the interventions that directly followed precleaning were evaluated for their effect on desorption of adhering bacteria (Fig. 1). Additionally, the viability of the remaining adhering bacteria after precleaning and the subsequent interventions was assessed.

### Effect of precleaning

After 30 minutes of bacterial adhesion, a mean of 1.66 × 10^6^ bacteria/cm^2^ (95% CI, 1.45–1.86 × 10^6^) adhered on FEP (Appendix 2). Precleaning with NDMCF was compared to precleaning with PBS, and both showed desorption of adhering bacteria. Precleaning with NDMCF resulted in a mean desorption of 0.60 × 10^6^ bacteria/cm^2^ (95% CI, 0.45–0.76 × 10^6^) and PBS in 0.87 × 10^6^ bacteria/cm^2^ (95% CI, 0.32–1.42 × 10^6^), leading to 64% (95% CI, 55%–73%) and 42% (95% CI, 5%–78%) reductions, respectively. The difference between flushing with NDMCF or PBS was not significant.

### Effect of precleaning plus interventions

**Fig. 2.**
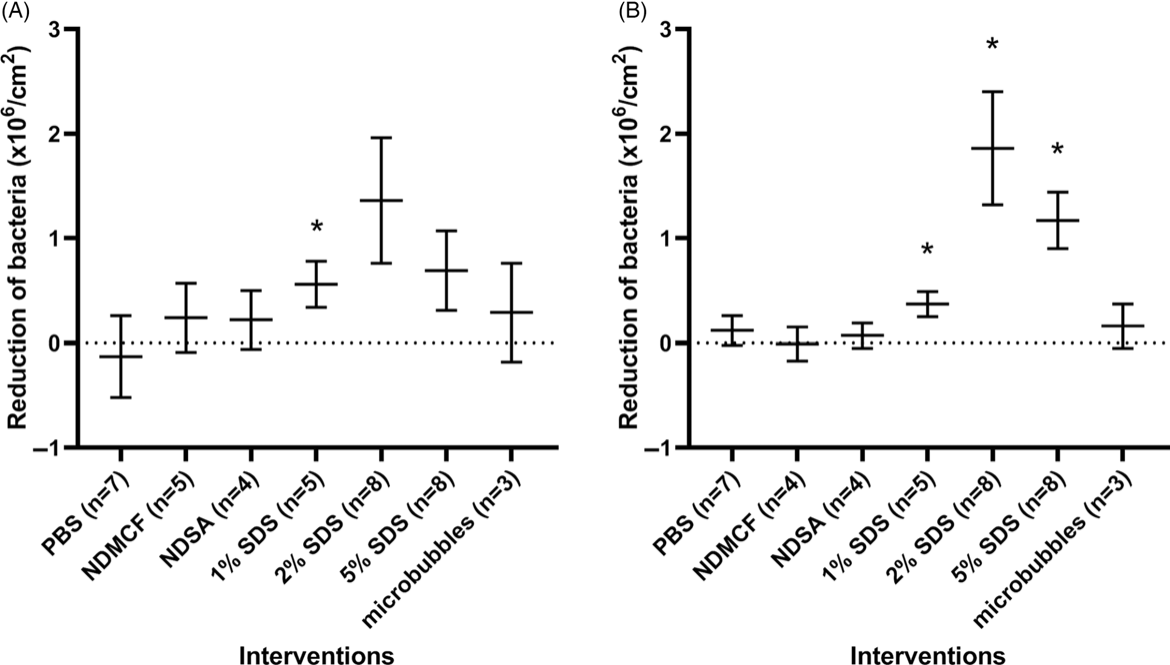
Desorption of adhering bacteria during interventions with 2 hours of flow compared with flow chambers left empty for 2 hours. (A) The difference between the mean number of adhering bacteria prior to precleaning compared to the number of adhering bacteria after an intervention. (B) The difference between the mean number of adhering bacteria after precleaning compared to the number of adhering bacteria after intervention. The dotted line represents no intervention. *P < .05 was considered significant.

**Fig. 3.**
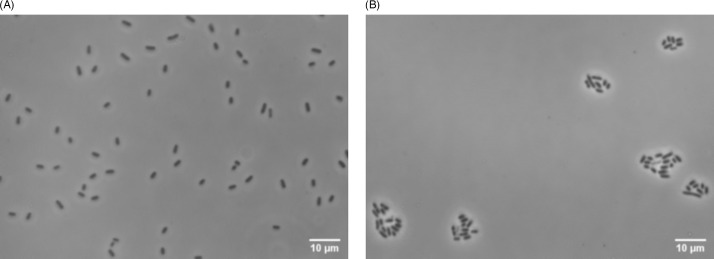
Images of adhering *K. pneumoniae* to FEP. (A) Bacteria randomly adhering on FEP before application of microbubbles. (B) Clustering of adhering bacteria after application of microbubbles.

The difference between the mean number of adhering bacteria prior to precleaning and that of adhering bacteria after the interventions were compared to no intervention (ie, flow chambers that were left empty for 2 hours after 30 minutes bacterial adhesion). The only interventions that caused desorption were those containing SDS 1%, 2%, or 5%. Only 1% SDS showed significant desorption (Fig. 2A). PBS without added compounds showed no desorption of bacteria. Interestingly, no desorption was observed with NDMCF or NDSA. Microbubbles also showed no bacterial desorption but, in contrast to other interventions, resulted in clustering of bacteria on the FEP (Fig. 3).

### Effect of interventions without precleaning

Bacterial desorption during the 2 hours interventions only was also analyzed by excluding desorption that had occurred during precleaning. The mean number of adhering bacteria after precleaning was 1.07 × 10^6^ bacteria/cm^2^ (95% CI, 0.83–1.31 × 10^6^). The difference between the mean numbers of adhering bacteria after precleaning and after the interventions were compared to that of flow chambers left empty for 2 hours (Fig. 2B). This analysis revealed that only interventions using SDS resulted in significant desorption of the number of adhering bacteria. A 1% SDS solution resulted in a 91% (95% CI, 84 %–98%) desorption compared to the mean number of adhering bacteria directly after precleaning. The SDS 2% and 5% experiments showed similar results, with 88% (95% CI, 80%–96%) and 98% reductions (95% CI, 96%–99%), respectively. No concentration-dependent effect of SDS on bacterial desorption was observed.

### Effect of interventions on viability of remaining adhering bacteria

The viability assay of the bacteria adhering on FEP after the interventions revealed that SDS and NDSA showed significant reductions of the remaining viable bacteria (Table 1). The MIC and MBC for NDMCF were 0.4 and 0.7 μg/mL. The MIC and MBC for NDSA were both 5 μg/mL. The MIC and MBC for SDS were 2.5 and >20 μg/mL.

**Table 1.** Remaining Adhering Viable Bacteria on FEP After the Interventions

Interventions (2-h flow)	No.	<30 CFU/cm^2^	30–300 CFU/cm^2^	>300 CFU/cm^2^	*P* Value^a^
PBS, %	5	0	20	80	.3571
NDMCF, %	3	0	33	67	.2500
NDSA, %	4	50	25	25	.0140
1% SDS, %	5	0	60	40	.0275
2% SDS, %	4	0	100	0	.0014
5% SDS, %	4	0	100	0	.0014
Microbubbles, %	3	0	0	100	>.9999

Note. PBS, phosphate-buffered saline; NDMCF, Neodisher Mediclean Forte; NDSA, Neodisher Septo Active; SDS, sodium dodecyl sulfate.

a The viability results of the interventions were compared to that of flow chambers left empty for 2 h. There were ten experiments with flow chambers left empty and all had >300 CFU/cm^2^ of growth after 2 h. *P* < .05 was considered significant.

## Discussion

We have described the novel dynamic flow model we used to model and evaluate an additional transport cleaning step between precleaning and manual cleaning in the reprocessing procedure. Different interventions were investigated for efficacy during this additional transport cleaning step.

A parallel plate flow chamber with an FEP bottom plate was used to mimic the internal lumen of a duodenoscope. The FEP bottom plate allowed real-time evaluation of the contamination rate during reprocessing, which is an advantage compared to other models (Table 2). This novel model for duodenoscope reprocessing was evaluated using a *K. pneumoniae* strain that survived standard reprocessing and led to a duodenoscopy-associated outbreak.^17^

**Table 2.** Duodenoscope Contamination Model Characteristics

Principal Component	Substratum	Advantage	Disadvantage	References
Pegs	PTFE	Easy application	No flow	da Costa Luciano et al^11^ da Costa Luciano et al^12^
Tubes	PTFE	Flow	No direct observation	Alfa et al^13^ Alfa et al^14^
Flow chamber	FEP	Flow direct observation inside lumen	Adhesion might be influenced because of different chemical composition	Bakker et al^15^ Kaper et al^16^

Note. PTFE, polytetrafluoroethylene; FEP, fluorinated ethylene propylene.

The interventions demonstrated that 2 hours of flow with an SDS solution removed adhering bacteria. SDS did not show a concentration-dependent effect because interventions using SDS always reached the detection limit regardless of the concentration used. After treatment with 1% SDS 5 × 10^4^ (95% CI, 1–9 × 10^4^) bacteria/cm^2^ remained on the FEP surface, which was 10-fold lower than an FEP surface without an intervention exposure. A bactericidal step remains warranted for the remaining bacteria on the surface.

Rinsing the contaminated duodenoscope during transport from the procedure room to the reprocessing facility with a 1% SDS solution would reduce the number of adhering bacteria in the duodenoscope channel prior to manual cleaning. Application of a device during the transport of the duodenoscope is feasible; however, a closed system is needed to avoid leakage and contact with air because SDS is a strong detergent and will form large amounts of foam when in contact with air.^20^

The possible health risks associated with the use of 1% SDS are limited given that toxicity after ingestion does not exceed the toxicity of table salt and SDS is not a carcinogen.^21^ An SDS concentration of <0.1% can be considered nonirritating to eyes, and although SDS at a concentration >2% can be irritating to skin, SDS is widely used as a foaming chemical in cosmetic products such as toothpaste and shampoo.^21,22^ Furthermore, duodenoscopes need to undergo high-level disinfection, which requires rinsing of the duodenoscope channels with water to remove the toxic chemicals used for high-level disinfection. Both application of high-level disinfection chemicals and rinsing afterward will result in a negligible amount of SDS remaining.^21,22^

Using SDS as an additional step in reprocessing would be interesting given that almost all reprocessing chemicals focus on their bactericidal properties and not on their ability to remove bacteria. Perhaps applying both in the same reprocessing procedure can be synergistic. Several chemicals used for high-level disinfection, such as glutaraldehyde and even some formulations with peracetic acid, can fixate proteins and thereby lead to bacterial accumulation in duodenoscope channels.^23,24^ Therefore, removal of debris and bacteria during manual cleaning is essential for the effectivity of high-level disinfection, and SDS could be an excellent addition in this regard, especially given its ability to disrupt biofilms.^25^ Further research will be needed to evaluate the effect of SDS on bacteria in conjunction with organic debris.

This study confirms that precleaning is essential for desorption of adhering bacteria, lowering the amount of adhering bacteria by 64%. Therefore, flushing a duodenoscope directly after use remains an essential part of reprocessing. No added benefit was seen from using NDMCF compared to PBS, which raises the question of whether detergents add to the removal of bacteria during precleaning. Application of NDMCF as intervention did not result in additional removal of adhering bacteria, and NDMCF only has a bactericidal effect with a concentration >20 times the maximum advised concentration of 1%.^26^ NDSA does have a strong bactericidal effect because it contains 0.15% peracetic acid. Applying 20 g/L NDSA for 15 minutes should be bactericidal, and when applied for 60 minutes, its effect should even be sporicidal, according to the manufacturer.^27^ Interestingly, when 20 g/L NDSA was applied in our experiment for 2 hours, viable bacteria could be recovered in 50% of the samples (Table 1). Furthermore, no significant desorption was observed with NDSA, meaning that a high number of dead bacteria remained in the lumen after application of NDSA. These dead bacteria could function as a growth medium for future microorganisms. Microbubbles, as applied in this study, did not add to the removal of adhering bacteria, in contrast to other studies that showed reduction of adhering bacteria with microbubbles.^28,29^ Currently, we cannot conclude that microbubbles are unsuited for bacterial removal in duodenoscopes because microbubble generation can be performed with a broad set of parameters, which was beyond the scope of this study.^28–30^ An important factor for desorption of adhering bacteria with microbubbles is flow rate, which was not evaluated in this study.^31^

The novel dynamic flow model for evaluation of duodenoscope contamination and reprocessing showed that precleaning is an essential part of duodenoscope reprocessing and that an SDS solution can be used to desorb adhering bacteria, making SDS a potential valuable application for duodenoscope reprocessing. The lack of effect by commercially available reprocessing chemicals is striking and warrants further investigation.

## Supplementary material

For supplementary material accompanying this paper visit https://doi.org/10.1017/ash.2022.294. click here to view supplementary material
