# Supplementary material for: A dynamic flow model mimicking duodenoscope reprocessing after bacterial contamination for translational research
Source: Antimicrob Steward Healthc Epidemiol. 2022 Sep 13;2(1):e153. doi: 10.1017/ash.2022.294 (PMC9726593; doi:10.1017/ash.2022.294)
Supplement: Supplementary file 1 [file ashsup.zip › S2732494X22002947sup002.docx]

**Supplemental material 2.** Number of adhering bacteria after pre-cleaning and different interventions for 2h.

|  |  |  | prior to pre-cleaning after 30 min bacterial adhesion  (1x10^6^ /cm^2^) | | |  | after pre-cleaning without additional intervention  (1x10^6^ /cm^2^) | | |  | after pre-cleaning and an additional intervention  (1x10^6^ /cm^2^) | | |
| --- | --- | --- | --- | --- | --- | --- | --- | --- | --- | --- | --- | --- | --- |
|  |  |  | mean | SD | 95% CI |  | mean | SD | 95% CI |  | mean | SD | 95% CI |
| Flow with PBS | (n=7) |  | 1.66 | 0.53 | 1.17 - 2.15 |  | 1.17 | 0.50 | 0.71 - 1.64 |  | 0.88 | 0.65 | 0.28 - 1.48 |
| Flow with NDMCF | (n=5) |  | 1.85 | 0.64 | 1.05 - 2.65 |  | 0.77 | 0.47 | 0.02 - 1.52 |  | 0.70 | 0.31 | 0.32 - 1.09 |
| Flow with NDSA | (n=4) |  | 1.53 | 0.23 | 1.18 - 1.89 |  | 0.64 | 0.20 | 0.32 -0.95 |  | 0.40 | 0.36 | -0.16 - 0.97 |
| Flow with 1% SDS | (n=5) |  | 1.52 | 0.37 | 1.06 - 1.98 |  | 0.58 | 0.20 | 0.33 - 0.83 |  | 0.05 | 0.03 | 0.01 - 0.09 |
| Flow with 2% SDS | (n=8) |  | 1.78 | 1.06 | 0.89 - 2.66 |  | 1.52 | 0.79 | 0.87 - 2.18 |  | 0.18 | 0.15 | 0.06 - 0.31 |
| Flow with 5% SDS | (n=8) |  | 2.33 | 1.63 | 0.97 - 3.69 |  | 2.08 | 1.47 | 0.85 - 3.31 |  | 0.05 | 0.04 | 0.02 - 0.09 |
| Flow with microbubbles | (n=3) |  | 1.50 | 0.78 | -0.42 - 3.43 |  | 0.64 | 0.35 | -0.23 -1.50 |  | 0.30 | 0.02 | 0.25 - 0.36 |
| No intervention* | (n=11) |  | 1.33 | 0.40 | 1.07 - 1.60 |  | 0.59 | 0.31 | 0.38 - 0.80 |  | 0.42 | 0.23 | 0.27 - 0.57 |

*Flow chambers that were subjected to NDMCF flush during pre-cleaning and afterwards were left with residual moisture for 2h instead of undergoing one of the interventions
