# Supplementary material for: A dynamic flow model mimicking duodenoscope reprocessing after bacterial contamination for translational research
Source: Antimicrob Steward Healthc Epidemiol. 2022 Sep 13;2(1):e153. doi: 10.1017/ash.2022.294 (PMC9726593; doi:10.1017/ash.2022.294)
Supplement: Supplementary file 1 [file ashsup.zip › S2732494X22002947sup001.docx]

A

B

**Supplemental material 1.** Overview of the flow chamber. A: cross section of the flow chamber. Black arrows indicate the flow through the chamber. Both the top and bottom plate have and a glass microscopy slide (white bars) sealed with a rubber gasket (black dots). The thin bar below the central black arrow represents the FEP sheet on top of the bottom glass slide. The gray bars at the sides represent a PTFE gasket to determine the depth of the flow chamber. B: top view of several principal components. Above is the top plate with a cutout to allow light to pass through the chamber and connectors for the flow system. In the center both gaskets are shown. In black the rubber gasket that prevents leakage between the plate and the glass slide and in grey the PTFE gasket that determines the depth in the flow chamber. Below the bottom plate is shown with cutouts for the rubber gasket and the glass slide.
